# Arterio‐venous anastomoses in isolated, perfused rat lungs

**DOI:** 10.14814/phy2.13023

**Published:** 2016-11-08

**Authors:** Robert L. Conhaim, Gilad S. Segal, Kal E. Watson

**Affiliations:** ^1^ The William S. Middleton Memorial Veterans HospitalMadisonWisconsin; ^2^ Department of SurgeryUniversity of Wisconsin School of Medicine and Public HealthMadisonWisconsin

**Keywords:** Alveolar capillary, arterio‐venous anastomoses, pulmonary circulation, pulmonary microcirculation, sheet flow

## Abstract

Several studies have suggested that large‐diameter (>25 μm) arterio‐venous shunt pathways exist in the lungs of rats, dogs, and humans. We investigated the nature of these pathways by infusing specific‐diameter fluorescent latex particles (4, 7, 15, 30, or 50 μm) into isolated, ventilated rat lungs perfused at constant pressure. All lungs received the same mass of latex (5 mg), which resulted in infused particle numbers that ranged from 1.7 × 10^7^ 4 μm particles to 7.5 × 10^4^ 50 μm particles. Particles were infused over 2 min. We used a flow cytometer to count particle appearances in venous effluent samples collected every 0.5 min for 12 min from the start of particle infusion. Cumulative percentages of infused particles that appeared in the samples averaged 3.17 ± 2.46% for 4 μm diameter particles, but ranged from 0.01% to 0.17% for larger particles. Appearances of 4 μm particles followed a rapid upslope beginning at 30 sec followed by a more gradual downslope that lasted for up to 12 min. All other particle diameters also began to appear at 30 sec, but followed highly irregular time courses. Infusion of 7 and 15 μm particles caused transient but significant perfusate flow reductions, while infusion of all other diameters caused insignificant reductions in flow. We conclude that small numbers of bypass vessels exist that can accommodate particle diameters of 7‐to‐50 μm. We further conclude that our 4 μm particle data are consistent with a well‐developed network of serial and parallel perfusion pathways at the acinar level.

## Introduction

Several recent studies have suggested the arterio‐venous shunt pathways exist within the lungs of humans, baboons, dogs, and rats (Eldridge et al. 2004; Lovering et al. 2007, 2009; Bates et al. 2012, 2014b). Evidence for the existence of these pathways has been demonstrated by the ability of large‐diameter latex particles or labeled albumin to flow through these lungs (Lovering et al. 2007, 2009; Bates et al. 2014a).

We have considerable experience measuring trapping patterns of specific‐diameter fluorescent latex particles infused into the circulation of intact and isolated lungs (Conhaim and Rodenkirch 1996, 1998; Conhaim et al. 2003, 2006, 2008a, 2008b, 2008c, 2008d; Watson et al. 2012). We wanted to use this experience to investigate the nature of the arterio‐venous shunt pathways reported by others.

Our approach was to infuse particles of 4, 7, 15, 30, or 50 μmol/L diameter into the pulmonary circulation of isolated‐perfused rat lungs. We then measured the number of particles that appeared in lung venous effluent samples over time. We also obtained digital confocal images of particles trapped within dried samples of the lungs after perfusion, and quantified these images to obtain data on the distributions of the trapped particles within the pulmonary circulation.

Appearances of particles of any of these diameters in the venous outflow would be evidence for the existence of arterio‐venous shunt pathways. Furthermore, variations in venous concentrations and trapping patterns among particles of each diameter would provide insight into the organization of the pulmonary circulation and improve our understanding of how the entire cardiac output can flow through the lung at one‐sixth the resistance of the systemic arterial circulation. The goal of our studies was to use our methods to investigate these ideas.

We performed additional experiments to determine if small particles might have flowed through the same shunt pathways as the large particles. We did this by partially embolizing lungs with 7 μm particles. We then infused 15 μm particles and compared venous concentrations of these particles with those in lungs that were not pre‐embolized. If the 15 μm particles flowed through the same shunt pathways taken by larger particles, then pre‐embolization with 7 μm particles would not affect the venous concentrations of the 15 μm particles.

## Methods

We used methods that were approved by the animal research committee of our institution. We used lungs isolated from retired, male, breeder Sprague–Dawley rats (450–550 g), which we anesthetized using isoflurane and killed by exsanguination following heparin infusion (750 U/kg).

### Lung preparation

We removed the heart and lungs en bloc, placed them dorsal side down, and cannulated the trachea, pulmonary artery, and left atrium using polyethylene tubing (PE 200). The lungs were ventilated with air (25 breaths/min) at inflation and deflation pressures of 15 and 5 cm H_2_O, and warmed using an incandescent lamp, that produced lung surface temperatures of 33 ± 2°C. We perfused the lungs via the pulmonary artery using a buffered hetastarch solution (1.5% Hespan in PBS; B. Braun Medical, Inc, Bethlehem, PA). The perfusate was pumped from a beaker into an arterial reservoir equipped with an overflow. The reservoir meniscus (overflow outlet) was set 10 cm above the bottom (dorsal side) of the lung, which therefore provided a pulmonary artery perfusion pressure of 10 cm H_2_O. The venous cannula was set level with the dorsal (bottom) side of the lung (*P*_ven_ = 0 cm H_2_O).

### Particle infusion

When perfusate flows were stable, after approximately 20 min, we infused 5 mg of fluorescent latex particles (Bangs Laboratories, Fishers, IN), suspended in 3 mL of perfusion solution, into the pulmonary arterial catheter over a period of two minutes. We added 0.5% albumin to the particle solution before suspension to coat the particles and neutralize their surface charge. This prevented them from clumping in the presence of saline ions in the perfusate buffer (Conhaim et al. 2003). Pulmonary artery pressures remained constant during particle infusion because the lungs were perfused from an overflow‐equipped reservoir. Infused particles were either of 4, 7, 15, 30, or 50 μm diameter. Particle diameters varied by about 7% for the smallest particles, to about 4% for the largest ones. Because all lungs received the same mass of latex, the number of particles infused was inversely proportional to the particle diameter (Table 1).

**Table 1:** Numbers of particles of each diameter infused into isolated, perfused rat lungs, and the percentage of the infused particles that appeared in the lungs' venous outflows. Each lung received 5 mg of latex, which resulted in the average number of infused particles shown. Particle concentrations were measured using a flow cytometer. A total of six lungs were prepared for particles of each diameter

Particle diam, μm	Numbers of particles infused	Percentage of infused particles that appeared in lung venous outflows (% of Infused)
4	1.7 × 10^7^	3.17 ± 2.46^a^
7	9.5 × 10^6^	0.04 ± 0.03
15	1.1 × 10^6^	0.04 ± 0.05
30	1.9 × 10^5^	0.01 ± 0.01
50	8.5 × 10^4^	0.17 ± 0.22

a Significant difference from all other particle diameters (*P* < 0.05).

At the moment we began particle injection, we placed a tube beneath the venous cannula to begin incremental collection of the venous effluent. We replaced the tube after 30 sec and repeated this at 30 sec intervals for 12 min. We used a flow cytometer (MACSQuant Analyzer 10; Miltenyl Biotec, Cologne, Germany) to measure the number of particles infused, and the numbers of particles in each of the venous effluent samples collected from each lung. We summed the particle counts from the effluent samples and expressed these as a fraction of the total number of particles infused. The perfusate volume in each tube was a measure of the perfusate flow during these timed collections.

When the particle collections were complete (12 min after the start of particle infusion), we clamped the arterial and venous cannulas, inflated the lungs to 20 cm H_2_O using compressed air, and maintained the lungs at this pressure for 2 days to allow them to dehydrate. We then sliced sagittal a section from the left or right hilum of each dried lung, and placed this onto the stage of a confocal microscope (Leica TCS‐LSI, Leica Microscopes, Hicksvilled, NY) where we obtained digital images (2.1 × 2.1 mm; 512 × 512 pixels; 4 per section) of the trapping patterns of the fluorescent particles within each lung.

### Particle‐to‐particle distance analysis

We used image analysis methods to quantify distances between individual particles or particle clumps in each confocal image. A clump of particles was treated as a single particle for these measurements. These values are a measure of the population densities of the vessels in which particles became trapped. We began by using public domain software (ImageJ, National Institutes of Health) to obtain the x‐y address of each particle within each image. We used these particle x‐y addresses to compute a distance matrix that consisted of the Euclidean distance from each particle or particle cluster to its nearest adjacent particle or cluster within each image. These values were averaged for all images obtained from each lung, and the mean of these within‐lung averages were used to calculate the average particle‐to‐particle distances for particles of each diameter. We have used these methods previously (Conhaim et al. 2008a).

### Embolization studies

We prepared four lungs in which we infused enough 7 μm diameter particles to cause perfusate flows to decline by 50% from pre‐particle infusion baseline. We then infused 5 mg of 15 μm diameter particles into these lungs, and measured the concentrations of 15 μm particles that appeared in the lungs' venous outflows. The goal of these studies was to determine if pre‐embolization with 7 μm particles changed the venous 15 μm diameter particle concentration or particle‐to‐particle distances, compared to lungs infused with 15 μm particles that were not pre‐emoblized.

### Statistics

Results are expressed as mean ± SD. A total of six lungs were prepared for particles of each diameter. Statistical comparisons were conducted using one‐way analysis of variance, and Fisher's least significant difference post hoc test to determine if differences were significant. We used this method to compare baseline flows with perfusate flows at each time‐point after the start of particle infusion for lungs perfused with particles of the same diameter. Differences were considered to be significant at *P* ≤ 0.05.

## Results

### Perfusate flows

**Figure 1:**
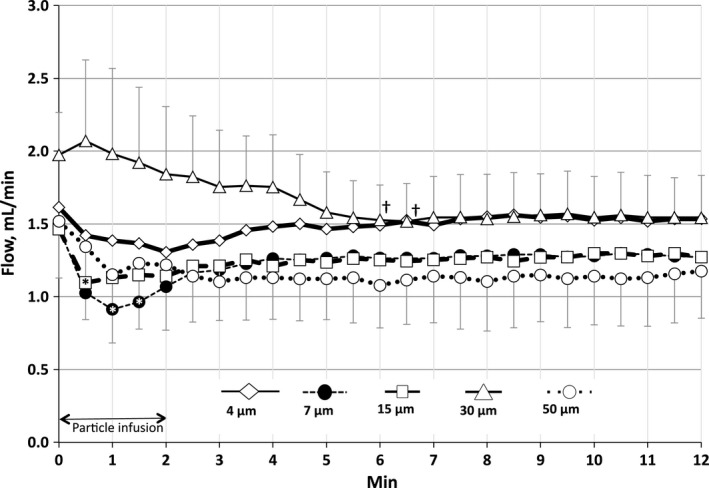
Perfusate flows after infusion of particles of each diameter (mean ± SD). Particle infusion began at time zero and continued for 2 min. Flows decreased significantly for 7 and 15 μm diameter particles at 1–2 min after particle infusion began (asterisks; *P* < 0.05). Flows also decreased transiently for the 30 μm particles at 6–6.5 min after infusion of those particles began (daggers).

Perfusate flows (Fig. 1) averaged 1.6 ± 0.2 mL/min in all lungs before particle infusion (0 min), and were not significantly different among particle diameter groups at this time. At 1–2 min after the start of infusion, flows fell significantly for the 7 and 15 μm diameter groups, although flows in these groups returned toward baseline after infusion. Flows declined insignificantly for the others.

### Venous particle concentrations

Particles of all diameters were able to flow through the lungs (Table 1). Venous particle concentrations, expressed as percentages of the numbers of particles infused, were small for particles larger than 4 μm (0.01% to 0.17%). An average of 3.17 ± 2.46% of the infused 4 μm particles (about 0.5 million particles) flowed through the lungs. This was significantly greater than percentage of infused particles of each of the larger diameters that flowed through.

**Figure 2:**
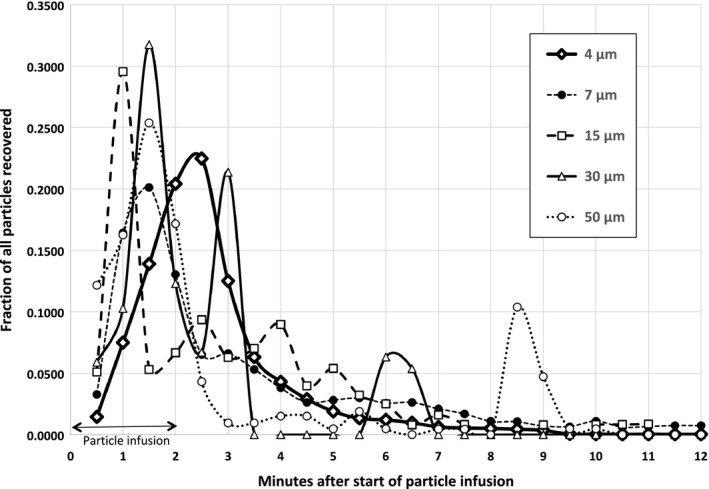
Appearance times of particles of each diameter in the venous effluent after start of particle infusions. *Y*‐axis values are the fractions of the total number of particles recovered that appeared in the venous outflow in each 30 sec sample. Totals = 1.0 for each diameter. *Y*‐axis error bars (not shown) are equivalent to the standard deviation values shown in Table 1. Times at which maximum concentration occurred were not significantly different among particles of all diameters.

Appearances of the particles within the venous outflow samples over time, expressed as a fraction of the total number of particles of each diameter that were infused, are shown in Figure 2. Particles of all diameters appeared in the first (30 sec) venous sample obtained after the start of particle infusion. The 4 μm particles appeared in an approximately uniform pattern. Appearances of the larger diameter particles were more irregular.

### Particle‐to‐particle distances

**Figure 3:**
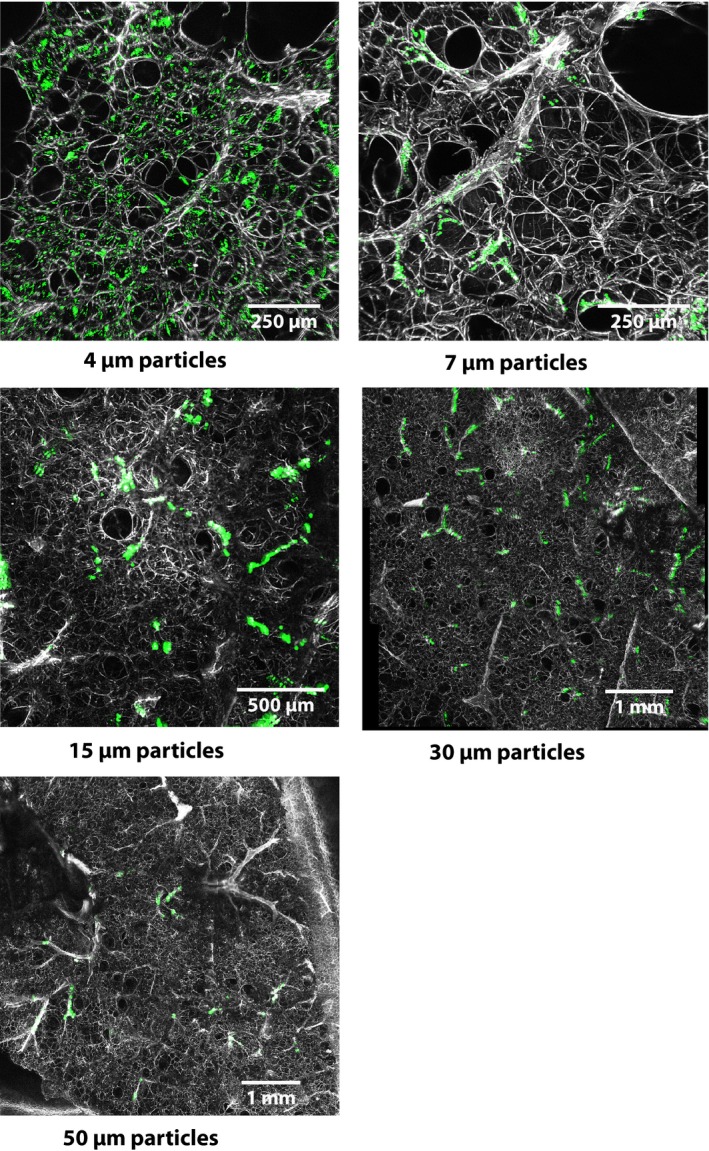
Trapping patterns of fluorescent latex particles (green) of diameters shown, in isolated rat lungs. Lung parenchyma appears gray. Average distances among these particles are shown in Figure 4.

**Figure 4:**
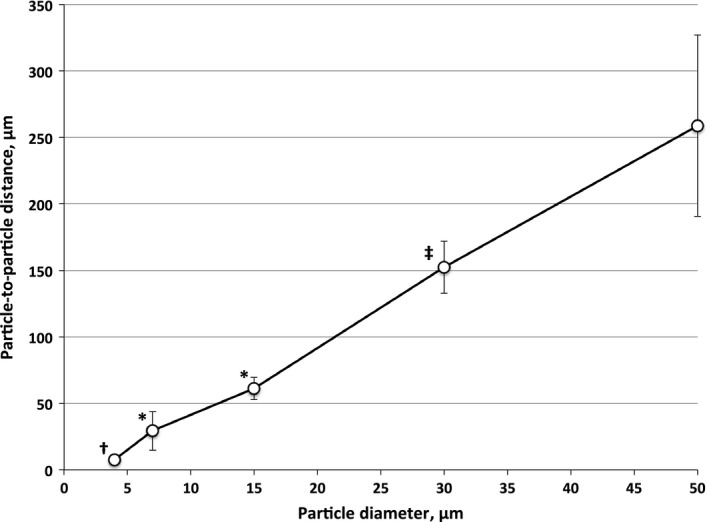
Average distance (μm) from one particle or particle cluster to the nearest neighboring particle in lung confocal images (mean ± SD) like those shown in Figure 3. ^**†**^Significant difference from 15 μm and larger particles (*P* < 0.05); ^*^Significant difference from 30 and 50 μm particles (*P* < 0.05); ^**‡**^Significant difference from 50 μm diam. particles of (*P* < 0.05).

Example confocal images showing trapping patterns for particles of each diameter within the lung parenchyma are shown in Figure 3. Average distances among trapped individual particles or particle clumps measured within these images are shown in Figure 4. We found a linear relationship between particle diameters and the particle‐to‐particle distances. These distances are a gage of the vessel population densities in which particles of each diameter became trapped.

### Embolization studies

Embolizing lungs with 7 μm particles caused perfusate flows to decline from 2.4 ± 0.9 to 1.3 ± 0.5 mL/min (*P* < 0.05). Infusion of 15 μm particles into these lungs after embolization caused no significant additional changes in perfusate flows. Flows averaged 1.3 ± 0.1 mL/min during the 2 min 15 μm particle infusion period, and 1.2 ± 0.1 mL/min for 10 min thereafter. We were unable to detect any 15 μm particles in the venous outflows from these lungs.

Particle‐to‐particle distances among 15 μm particles in lungs pre‐embolized with 7 μm particles averaged 112.6 ± 38 μm compared to 61.3 ± 8.4 for 15 μm particles in lungs not pre‐embolized (*P* < 0.05) (Fig. 4).

## Discussion

We found that at least some particles of all diameters were able to flow through the circulations of isolated, perfused, rat lungs that had not been pre‐embolized. This suggests that some bypass vessels were present among all vessel populations that the particles could enter. Based on the venous particle concentrations, however (Table 1), the number of these bypass vessels was apparently quite small for vessels larger than those entered by 4 μm particles.

Our results provide no information about the identity or location of these bypass vessels. However, the effects of the particle infusions on perfusate flows, venous particle concentrations, and rates of appearance of the particles in the venous outflow provide some insight into the paths taken by particles of each diameter within the pulmonary circulation, as follows.

The effects of particle infusions on perfusate flows were not equal for all particle diameters. Infusion of 4 μm particles produced slight but insignificant reductions in flow (Fig. 1), while infusion of 7 and 15 μm diameter particles produced significant flow reductions during the first 1–2 min of infusion, after which flows returned toward pre‐infusion baseline. This was despite the fact that the number of 4 μm particles infused was nearly twice that of 7 μm particles, and 15‐fold greater than the number of 15 μm particles infused. Virtually, all of the 7 and 15 μm particles became trapped within the circulation because only 0.04% of particles of each of these diameters appeared in the venous outflow (Table 1). The significant reduction in flow during the first 1–2 min of the 2‐min particle infusion period for the 7 and 15 μm particles suggests that those particles partially obstructed arterial inflow during their entry into the circulation. However, once they became trapped, flows must have increased among the remaining unobstructed vessels, compared to flow among those vessels prior to particle infusion (Fig. 3). This is the only explanation for the return of perfusions toward baseline after particle infusions were completed. Flows after infusions were completed were insignificantly lower than preinfusion baseline.

Particles of all diameters began to appear in the venous samples of the unembolized lungs within 30 sec after the start of particle infusion (Fig. 2). Although few particles with diameters larger than 4 μm appeared (Table 1), the fact that these particles began to appear so quickly after the start of infusion suggests that their path length through the circulation was short. The appearance times of 4 μm particles followed a uniform, dye‐dilution type of pattern, while appearances of the larger particles were more erratic. This was despite the large numbers of 4 μm particles infused, and the comparatively large number of those particles that appeared in the venous outflow (Fig. 2, Table 1). The 4 μm particles that appeared in the venous outflow represented only 3% of the particles infused. The relatively leisurely rate at which these particles appeared in the venous outflow (Fig. 2), compared to rates for larger particles, suggests that the 4 μm particles may have taken a longer path through the parenchyma than the larger particles. Furthermore, the significantly higher venous particle concentration for the 4 μm particles suggests that the pathways for these particles were more abundant than those for larger particles (Table 1). We cannot rule out the possibility that some 4 μm particles may have entered the venous outflow after flowing through alveolar septal capillaries, which in rats have diameters of 5–6 μm (Short et al. 1996). These rigid latex particles cannot deform within capillaries as do red cells. However, if the particles had entered the venous outflow after flowing through the septal capillaries, we would have expected a greater fall in perfusate flows during and after particle infusion due to obstruction of the capillaries by the particles. The fact that 97% of the 4 μm particles remained trapped within the circulation (Table 1; Fig. 2), while perfusate flows declined insignificantly after particle infusion (Fig. 1), suggests that an extensive parallel perfusion pathway must exist within the microcirculation at the acinar level through which the particle‐free perfusate was apparently able to flow after the particle infusions were complete.

Average distance among trapped individual particles or particle clumps followed a linear relationship with particle diameter (Fig. 4). This relationship is an indicator of the number of vessels accessible to particles of each diameter, but it is not a direct measure of that vessel population because if particles had entered all such vessels, flow would have stopped. Rather, this relationship reflects the number of vessels in which particles became trapped, and is based on the number of particles infused. Infusion of more particles would have included more vessels and reduced the particle‐to‐particle distances, but would have also caused greater reductions in perfusate flow. All lungs received the same mass of latex, but not the same number of particles (Table 1).

Particle clumps suggest that individual particles serially embolized the vessels in which the clumps appear. The particles were not clumped before they were infused, so appearance of clumps implies that particles continued to flow into these vessels until they became completely obstructed.

Several years ago, Fung and Sobin suggested that alveolar septal perfusion could better thought of as “sheet flow,” rather than as flow through individual vessels (Fung and Sobin 1969). Their hypothesis was based on the idea that the alveolar septal capillaries were so short and so closely spaced that the notion of blood flow through a single tube did not accurately portray the nature of alveolar septal perfusion.

More recently, Clark and colleagues proposed a theoretical pulmonary microcirculation model that consisted of both serial and parallel microperfusion pathways among and within the pulmonary acini (Clark et al. 2010). Their model predicted significantly longer red cell transit times in the distal portion of the acinus compared to the proximal portion. Their model implies that Fung and Sobin's sheet flow hypothesis is not restricted to the alveolar septum, but may extend into and include the pulmonary acini.

We believe the results we obtained using 4 μm particles supports this hypothesis. We found the venous particle concentrations for these particles to be significantly higher than for larger particles, and also found the transit time for these particles through the circulation to be longer than for the larger particles. We also found that perfusate flows declined insignificantly after completion of the 4 μm particle infusions. These findings are consistent with a perfusion system that consists of both series and parallel perfusion pathways at the acinar level, as Clark and colleagues suggested.

Our lungs were ventilated, and we cannot say how particle flow through bypass vessels at the acinar level might have changed if the lungs had been perfused at constant inflation volume. Several years ago, Albert and colleagues showed that lung inflation increased the diameters of small, extra‐alveolar arterioles (0.2‐to‐1.3 mm diam.) in isolated dog lungs (Albert et al. 1993). If serial and parallel microperfusion pathways exist at the acinar level as Clark and colleagues hypothesize, then lung inflation and deflation might be expected to redistribute the theoretical sheet of blood flowing through these vessels in a way that would optimize blood arterialization.

Bates and colleagues infused 1.0 × 10^6^ 15 μm microspheres into isolated, perfused rat lungs and counted the total number that were able to enter the pulmonary venous outflow (Bates et al. 2012). By comparison, we infused 1.1 × 10^6^ 15 μm particles (Table 1). They found about 60% as many particles in the venous outflow of their lungs compared to the number we found. Their methods were similar to ours, but with subtle differences. Their particles were infused in 4, 1 mL boluses (250 000 particles/bolus) over 20 min, whereas ours were infused continuously in 3 mL over 2 min. Their lungs were ventilated at the same inspiratory and expiratory pressures as ours (15/5 cmH_2_O) but were ventilated at 30–45 breaths/min, compared to 25 breaths/min for ours. Their lungs were perfused at a pulmonary artery pressure of 20 cmH2O, compared to 10 cm H_2_O for ours. Accordingly, they obtained perfusate flows of 8.9 mL/min, compared to 1.6 mL/min for ours. They used a fluorescence microscope to count the total number of particles on a piece of filter paper through which the venous effluent had been vacuum filtered. They recovered an average of seventy 15 μm spheres per lung, or about 60% as many particles as we recovered. A possible explanation for the differences in our findings is that their method of infusing particles in boluses rather than continuously, caused particle trapping and partial obstruction of the shunt pathways, so that fewer particles flowed through their lungs.

Bates and colleagues also found that hypoxia (P_a_O_2_ < 30 mmHg) increased the passage of 15 μm spheres through the lungs of intact rats, but hypoxia had no effect on the passage of those particles through isolated rat lungs (Bates et al. 2012). They concluded that hypoxia recruits intra‐pulmonary arterio‐venous anastomoses in intact rats but not isolated rat lungs. An important difference between intact and isolated lungs is that intact lungs are ventilated by negative intrapleural pressures, while isolated lungs are inflated by positive airway pressures. Indeed, hypoxia to the level Bates and colleague employed would have increased the rate and depth of breathing, and produced intrapleural pressures markedly more subatmospheric than those present during normoxic breathing. This could have markedly expanded the diameters of the bypass vessels, due to increased parenchymal traction on those vessels caused by increased lung expansion. Hypoxia would not have this effect in isolated lungs mechanically ventilated with positive pressure. This is a possible explanation for their finding that hypoxia recruited bypass vessels in intact but not isolated rat lungs.

Greater subatmospheric pressures associated with hyperpnea could also help explain why arterio‐venous anastamoses appear in the lungs of humans and dogs during exercise (Eldridge et al. 2004; Stickland et al. 2007; Lovering et al. 2009).

It could be argued that the lung contains no small vessel shunt pathways, but only large ones as described previously by Eldridge and colleagues. That is, all particles smaller than 50 μm diameter that we found in venous outflows may have actually flowed through the same large‐diameter shunt pathways that the 50 μm particles flowed through. The main argument against this idea is the results we obtained when we infused 15 μm particles into lungs that were first embolized with 7 μm particles. We found that 15 μm particles were unable to flow through these lungs, unlike non‐embolized lungs in which we found that 15 μm venous particle concentrations were about 0.04% of the particles infused (Table 1). This suggests that embolization with 7 μm particles blocked the shunt pathways previously available to 15 μm particles. The 7 μm particles are obviously smaller than pathways through which 15 μm particles flowed, but clumps of the 7 μm particles (Fig. 3) may have blocked the entrances to the bypass channels through which small numbers of 15 μm particles otherwise would have flowed if the 7 μm particles had not been present. If 15 μm particles flowed through the same shunt pathways as the 50 μm particles, then 15 μm venous particle concentrations in embolized lungs would have been similar to those in lungs not embolized (0.04%). We also found that 15 μm particle‐to‐particle distances in embolized lungs were significantly greater than in non‐embolized lungs. We believe this shows that fewer vascular pathways were available to these particles as a result of the pre‐embolization. Perfusate flows did not change after infusion of the 15 μm particles, despite the apparent reduction in available vascular pathways, which we believe is consistent with the idea of both series and parallel perfusion pathways at the acinar level.

In summary, we found that particles of 50, 30, 15, 7, and 4 μm diameter were able to flow through isolated rat lungs. However, we believe the significantly larger venous concentration of 4 μm particles, along with the lack of significant flow reduction as these particles were infused, is consistent with the idea of an extensive network of series and parallel perfusion pathways at the acinar level.

## Conflict of Interest

Supported by the Veterans Health Administration, Office of Research and Development, Department of Veterans Affairs, Biomedical Laboratory Research and Development Service. The contents do not represent the views of the Department of Veterans Affairs or the United States Government.
